# Assessing Weather-Yield Relationships in Rice at Local Scale Using Data Mining Approaches

**DOI:** 10.1371/journal.pone.0161620

**Published:** 2016-08-25

**Authors:** Sylvain Delerce, Hugo Dorado, Alexandre Grillon, Maria Camila Rebolledo, Steven D. Prager, Victor Hugo Patiño, Gabriel Garcés Varón, Daniel Jiménez

**Affiliations:** 1 Decision and Policy Analysis (DAPA), International Center for Tropical Agriculture (CIAT), Cali, Colombia; 2 Haute Ecole d'Ingénierie et de Gestion du Canton de Vaud (HEIG-VD), Yverdon-les-bains, Switzerland; 3 Agrobiodiversity Rice department, International Center for Tropical Agriculture (CIAT), Cali, Colombia; 4 Colombian National Rice Growers Federation (Fedearroz), Bogotá, Colombia; Fujian Agriculture and Forestry University, CHINA

## Abstract

Seasonal and inter-annual climate variability have become important issues for farmers, and climate change has been shown to increase them. Simultaneously farmers and agricultural organizations are increasingly collecting observational data about in situ crop performance. Agriculture thus needs new tools to cope with changing environmental conditions and to take advantage of these data. Data mining techniques make it possible to extract embedded knowledge associated with farmer experiences from these large observational datasets in order to identify best practices for adapting to climate variability. We introduce new approaches through a case study on irrigated and rainfed rice in Colombia. Preexisting observational datasets of commercial harvest records were combined with in situ daily weather series. Using Conditional Inference Forest and clustering techniques, we assessed the relationships between climatic factors and crop yield variability at the local scale for specific cultivars and growth stages. The analysis showed clear relationships in the various location-cultivar combinations, with climatic factors explaining 6 to 46% of spatiotemporal variability in yield, and with crop responses to weather being non-linear and cultivar-specific. Climatic factors affected cultivars differently during each stage of development. For instance, one cultivar was affected by high nighttime temperatures in the reproductive stage but responded positively to accumulated solar radiation during the ripening stage. Another was affected by high nighttime temperatures during both the vegetative and reproductive stages. Clustering of the weather patterns corresponding to individual cropping events revealed different groups of weather patterns for irrigated and rainfed systems with contrasting yield levels. Best-suited cultivars were identified for some weather patterns, making weather-site-specific recommendations possible. This study illustrates the potential of data mining for adding value to existing observational data in agriculture by allowing embedded knowledge to be quickly leveraged. It generates site-specific information on cultivar response to climatic factors and supports on-farm management decisions for adaptation to climate variability.

## 1. Introduction

Decision making by farmers on what crop to grow, where, and when, necessarily takes into account numerous factors, many of which are highly variable [1]. Among them, climatic conditions directly affect the performance of crops [2] and are therefore of principal importance. Recent research demonstrates the role of climate change in causing shifts in seasonal/inter-annual climate variability (hereafter referred to as climate variability [3]), with weather patterns becoming less consistent with farmers’ experience and crops experiencing more extreme climatic events [4,5]. In the tropics, sea surface temperature of the Niño 3 and Niño-3.4 regions in the Pacific Ocean, a key driving force of climate variability [6,7], is trending toward increased inter-annual variability with simulations of future climates forced by higher greenhouse gas concentrations suggesting that this trend will continue [8]. There is thus little doubt that climate variability will continue to present challenges for agriculture in the region.

This context is reflected in increased variability in crop yields [9,10] and greater uncertainty for farming related businesses [11]. In Colombia, for example, the national average yield for irrigated rice has dropped from approximately 6,000 kg·ha^-1^ to 5,000 kg·ha^-1^ over the last five years [12,13], with local agronomists hypothesizing climate variability as the main cause. Rice is a staple crop in the country with per capita intake estimated at 37.7 kg·person^-1^·year^-1^ [14]. The socio-economic impacts of yield variability are further exacerbated when the impact on competitiveness is considered, especially since the US-Colombia free trade agreement went into effect in May 2012.

To counter the impacts of climate variability, understanding how different climatic factors affect rice yields is a key objective of the Colombian National Rice growers Federation (Fedearroz). Weather-yield relationships are dynamic and depend on a complex set of interactions between local biophysical conditions and crop management practices. Whilst previous global and continental scale studies have successfully characterized the impact of climate variability on yields [15–18], they have limited direct relevance to farm-level decisions as they do not provide information about specific weather-cultivar-yield relationships occurring at local scale. Indeed, of the numerous potential limiting factors that have been characterized in the literature for rice, farmers still need to know which factors actually limit productivity on their specific farm and in what order of relevance. Traditional experimental schemes for local multisite characterizations of these issues are expensive and take time. To support tactical farm-level decisions, more dynamic and specific studies are needed [19]. These studies should leverage site-specific knowledge through in situ daily weather and production records, and assess the influence on yields of more climatic variables than simply temperatures and precipitation.

Comprehensive assessments of the influence of climate variability on crop yields at local and regional scales can benefit from the use of disaggregated observational data. Characterizing yield, weather, soil and management factors, these data have the potential to improve the spatial and temporal resolution of the results of empirical modelling approaches. Examples where non-experimental data have already been shown to embody useful information include prediction of influenza spreading [20], real-time rainfall estimation [21], and medical cohort studies [22]. For agriculture, much of the data needed for such assessments are often already available as many agricultural organizations have been collecting information for decades for internal purposes without realizing that such data may hold significant latent value in unanticipated uses. Simultaneously, more and more data are being generated thanks to increasingly inexpensive sensor networks, remote sensing and the spread of information and communication technologies [23,24]. Nevertheless such data often remain underexploited as many organizations lack the tools to harness the information with reliability, rapidity and accuracy so as to generate timely recommendations.

Here, we leverage and further develop the concept of data-driven agronomy, an approach that, in contrast to traditional controlled experiments, combines observational data, agronomy, analytics, and the principles of operational research [25,26]. The approach relies on data collected on commercial cropping events that simultaneously capture a wide range of combinations of weather, soil and management conditions along with corresponding yields; these diverse datasets therefore capture the inherent variability in operating conditions of commercial farms [27].

While large farm-level observational datasets often do not meet the standards of traditional statistical methods, they open up the possibility of using data mining approaches to discover and analyze trends that may occur in agricultural systems. Machine learning techniques thus represent an alternative approach to allow the discovery of embedded knowledge that may be present in the data. Successful examples of these types of approaches to support agronomic decision-making include the use of both supervised and unsupervised artificial neural networks to model Andean Blackberry (*Rubus glaucus)* yields [26], and the use of mixed models to determine optimum growing conditions of Lulo (*Solanum quitoense)* [28]. Classification And Regression Trees (CART) offer a more interpretable scheme that has been used to analyze maize [29] and rice [27]. More recently, CART and CHi-squared Automatic Interaction Detection (CHAID) models were combined to analyze maize data [30]. Nevertheless, only a handful of studies have used observational datasets based on truly non-experimental crop data to train empirical models, including agricultural systems for a variety of food crops in tropical regions [25,26,28], along with cotton in more temperate regions [31].

In this research we introduce an approach based on observational data and data mining techniques to support decision making in rice agriculture. Our first objective was to analyze the role of climate variability as a limiting factor for large series of rice cropping events in two contrasting regions of Colombia. Our second objective was to assess the diversity of weather patterns under which rice was produced, in order to quantify their impact on yields and to identify best suited cultivars for each condition. The complete process of data preparation, the framework for selection of methods, the two-phase analysis, and its substantiation are presented below.

## 2. Materials and Methods

### 2.1. Study areas

**Fig 1:**
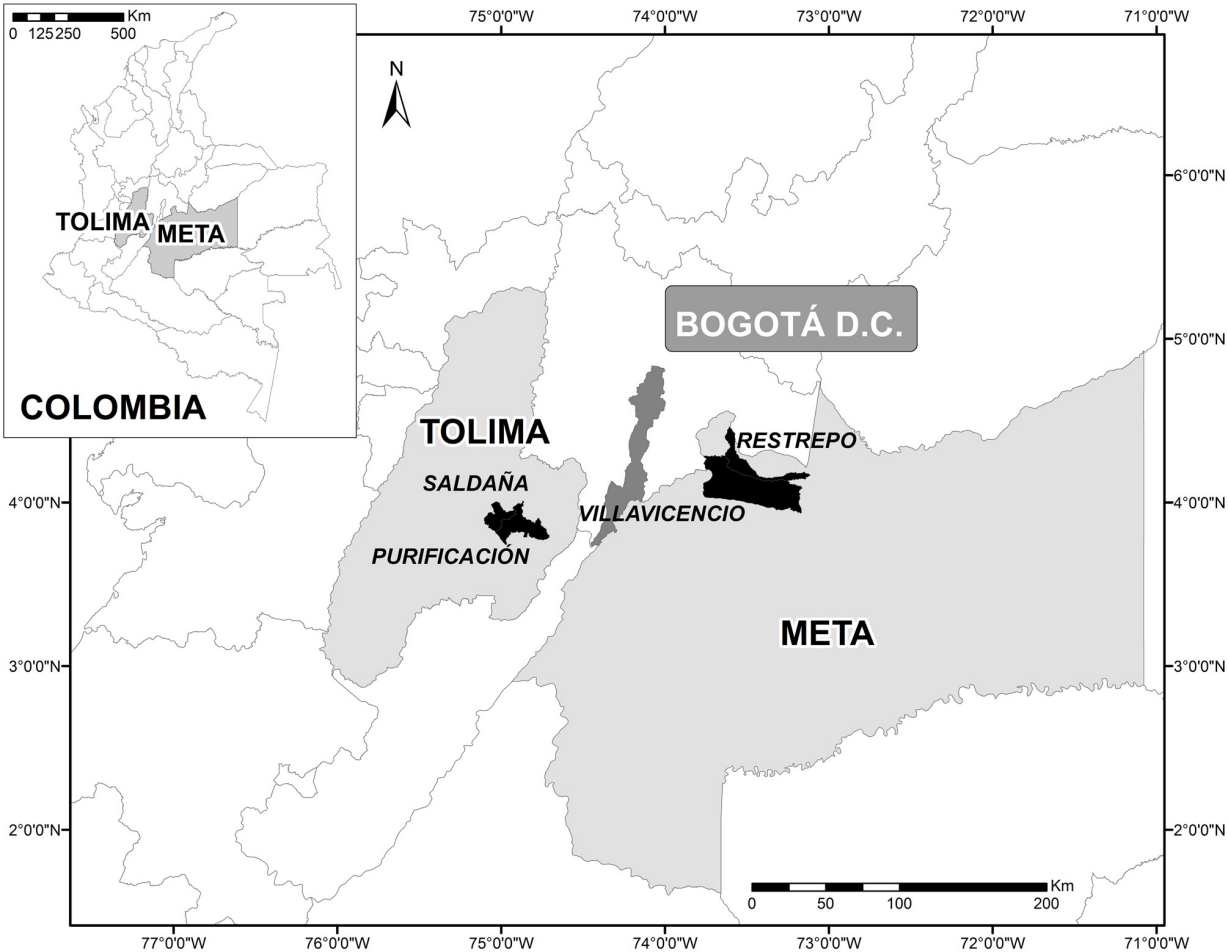
Location map of the study areas.

In 2014, Colombia produced approximately 1.69 million tons of paddy rice, of which 1.1 million tons (65%) were produced by lowland irrigated rice crops on 217,000 ha (58%), and 587,000 tons (35%) by rainfed rice on 156,000 ha (42%) [13]. This study focuses on two important, yet contrasting regions and cropping systems that account for more than 70% of the rice production in the country: the central department of Tolima (lowland irrigated rice) and a large area known as the “Llanos” or plains in the Meta department (rainfed rice) (Fig 1).

Tolima is the leading rice producing area in Colombia. The region is topographically varied and is located in the Magdalena river valley formed by the Andean mountain chain. Lowland irrigated rice is grown twice a year in the low flatlands at approximately 300 meters altitude. Average precipitation in Tolima is approximately 1,400 mm per year with a bimodal distribution: the first rainy season starts around the end of March/beginning of April, and the second one around the beginning of September. Average temperature remains relatively constant throughout the year at around 27°C. Soils are predominantly Inceptisols with fine texture, slightly acidic to neutral pH, and contain moderate amounts of organic matter [32]. Within Tolima, we focused on the localities of Saldaña and Purificación, located in the southeast of the department (Fig 1), where an average of 12,000 ha of rice are grown in each semester. Farms are relatively small, with an average area of 6 ha, and are typically family run enterprises with medium levels of technology adoption. Unless otherwise noted, hereafter we use the name Saldaña to refer to both municipalities.

The second area is commonly called “Los Llanos Orientales” and refers to the immense plains just east of the Andes. Soils of this region are mainly oxisols and to a lesser extent, ultisols. They tend to be more acidic, and with high levels of aluminum and compaction [33]. Although there is abundant rainfall with 2,000 to 3,500 mm per year following a monomodal distribution, little of this water benefits the rice crops due to uneven rainfall and the lack of irrigation infrastructure. The rainy season starts around the end of March/beginning of April. Average temperature remains almost constant throughout the year at around 26°C. These large plains have been traditionally used in extensive livestock production and oil palm tree plantations, with rice concentrated in areas closer to the piedmont. In the “Llanos”, rainfed rice is typically sown once a year, just before the start of the rainy season. Farmers tend to be entrepreneurial and typically rent the land each season depending on how they perceive the relative profitability of the crop for that year. We analyzed data from two municipalities in the department of Meta: Villavicencio and Restrepo (Fig 1), where 6,300 ha of rainfed rice are sown in the first semester. We use the name Villavicencio to refer to both of these localities hereafter.

### 2.2. Data sources and preprocessing

Non-experimental or observational data can come from many sources. Working with multiple sources of data can be challenging as the data are often highly disparate. Here, we construct a database of non-experimental observations from different sources, carefully addressing the challenges and opportunities associated with converting this kind of data into usable information.

#### 2.2.1. Commercial crop data

Fedearroz has been collecting information on commercial rice crops in the rice-producing areas of Colombia for a range of purposes, including yield monitoring and crop profitability studies. As a result, the Federation holds multiple datasets of varying quality, different sample sizes and at multiple levels of detail in terms of recorded variables. Not all the information is centralized yet, nor it all exist in digital form. For this study, we had access to a portion of this information, and used three different datasets. (a) The harvest monitoring dataset was the largest. Records in this dataset are based on large scale routine surveys in the regions designed to monitor production levels. This dataset includes cropping system, harvest date, location, cultivar and yield. (b) The national rice survey is a detailed biannual monitoring of a representative sample of rice farms in Colombia. This survey has been running since 1988 and includes many variables associated with management practices, costs, and production. An average of 130 rice fields are surveyed semiannually throughout the rice producing regions, with five visits to each. (c) Finally, sowing dates experiments are routinely run by Fedearroz each year to assess the influence of the sowing period on yields. Different sowing dates are tested under standard management to assess crop response. Although these are experimental data, we included them in the study because they bring additional temporal variability as a function of the unusual sowing dates. Table 1 summarizes the availability of data from each source in each of the localities as well as the time periods covered by the data.

**Table 1:** Summary of data sources for cropping events.

		Number of observations available	
Dataset	Years with available data for the study	In Saldaña (irrigated rice)	In Villavicencio (rainfed rice)
(a) Harvest monitoring	2007 to 2014	945	268
(b) National Rice Survey	2007 to 2012	95	28
(c) Sowing date experiments	2012 to 2013	200	79
**Total**		**1,240**	**375**

The data were standardized for consistent spelling and measurement units. In cases where data were sparse, incoherent or duplicated, the corresponding records were removed. The data were then merged into a new data frame that included only the variables in common among all of the three datasets, namely: municipality, cultivar, cropping system, sowing date, harvest date, and yield. When the sowing date was not available (80% of the data), we estimated it by subtracting 126 days from the harvest date in Saldaña, and 128 days in Villavicencio. Those durations were set according to the average crop cycle length of the remaining records with both sowing and harvest dates measured, and coincide with the typical duration of the rice crop cycle in the tropics [34,35] and in Colombia [36]. Exact harvest dates were available for all records, which guaranteed that the cropping events could be accurately located in time.

#### 2.2.2. Daily weather data at the station level

We obtained daily weather records from the National Institute of Hydrology, Meteorology, and Environmental Studies (known as IDEAM), and from one weather station of the Fedearroz agro-meteorological network. The five most important climatic variables for rice growth [35] were considered: minimum temperature (TM), maximum temperature (TX), precipitation (P), relative humidity (RH) and solar radiation (SR).

Stations were initially selected according to the availability of desired variables, quality of data, length of the available time series, and proportion of missing values. In each of the study areas, the set of selected stations was further screened based on: nearness to rice crops (<10 km), similar altitude level (±50m relative to the crops), and consistency in series between one another. The closest station to the rice fields was then selected, while the others served as supporting information for estimation of missing values.

Hourly records were converted to daily when more than 80% of the data was available; otherwise the day was considered a missing value. Each of the weather series was quality controlled following the World Meteorological Organization guidelines [37]. In Villavicencio none of the stations measured SR, but all of them had sunshine duration which corresponds to sunny hours per day. We calculated SR from sunshine duration using Angstrom’s equation suggested by FAO in [38]. This was done using the R package SIRAD [39]. In Saldaña, in order to reduce the proportion of missing values to be estimated, stations ID#15 from Fedearroz and ID#21135020 from IDEAM were merged as they had complementary recording periods and were very close to one another (approximately 7km distant).

Remaining missing values for TM, TX and P were then estimated by a weather generator based on Vector Autoregressive models (VARs) for multivariate time series using the R package RMAWGEN [40]. For RH and SR, a different method was used: a random forest model was trained using the R package randomForest [41] on a sample dataset using TM, TX and P as predictors to estimate missing values. The parameters for randomForest were: 800 trees (ntree = 800) with two predictors used at each node (mtry = 2). Once all the data were cleaned and missing values filled, we obtained one representative weather series for each locality. Finally we computed daily values of the average temperature (TA hereafter) ((TX-TM)/2) and the diurnal range (DR hereafter) (TX-TM).

The above procedures were implemented in Saldaña using data from a combination of two stations from IDEAM and one from Fedearroz (S1 Table). In Villavicencio, four stations from IDEAM were used (S2 Table). These weather data, in combination with the collected crop data thus served as the primary inputs for the analytic process.

#### 2.2.3. Data preparation

Our analysis unit was the cropping event as defined by Cock *et al*. [25]. A cropping event is the combination of, (i) a yield record at a given time in a specific field and, (ii) the records of everything that happened to that specific crop from sowing to harvest. The definition includes the characterization of environmental conditions, namely climate and soil, and of the management practices implemented by the farmer. Cropping events are unique in time and space, but similar events can occur in different places or in the same place at different times.

In this study we focus on climatic factors. We related each individual harvest record to the daily weather data corresponding to the period between sowing and harvesting dates, which corresponds to the weather pattern the crop effectively experienced. The seven previously described climatic variables, TX, TM, P, RH, SR, TA, and DR were used as the basis to characterize the cropping events.

Short cycle crops like rice have specific requirements and sensitivity to environmental factors in each growth stage that are directly related to the physiological processes occurring in the plant [35,42]. To understand plant response to climatic factors, we calculated specific indicators out of the raw weather data separately for each of the three growth stages as defined by Yoshida [35]: vegetative (VEG) from germination to initiation of panicle primordia, reproductive (REP) from panicle primordia initiation to heading, and ripening (RIP) from heading to maturity. For each cropping event the panicle initiation and flowering dates were estimated based on the local knowledge of agronomists and physiologists from Fedearroz, taking into account the differences between cultivars. They defined the typical durations of each growth stage for each cultivar on the basis of a 126 days cropping period. The estimated dates were then calculated for each cropping event according to the cultivar grown and adjusting those durations proportionally to the actual total growth period, to take into account shorter/longer cropping events. These dates served to split the crop growth period and the corresponding weather records into the three growth stages. Resulting variables are listed in Table 2 where each indicator appears three times, one for each growth stage. Temperature thresholds were adapted from Sánchez et al. [42].

**Table 2:** List of the variables used in the models.

Variable name	Meaning	Type	Unit
Cultivar	Cultivar that was grown	Categorical	
TX_Avg_VEG	Average maximum temperature in vegetative stage	Continuous	°C
TX_Avg_REP	Average maximum temperature in reproductive stage	Continuous	°C
TX_Avg_RIP	Average maximum temperature in ripening stage	Continuous	°C
TM_Avg_VEG	Average minimum temperature in vegetative stage	Continuous	°C
TM_Avg_REP	Average minimum temperature in reproductive stage	Continuous	°C
TM_Avg_RIP	Average minimum temperature in ripening stage	Continuous	°C
TA_Avg_VEG	Average temperature in vegetative stage	Continuous	°C
TA_Avg_REP	Average temperature in reproductive stage	Continuous	°C
TA_Avg_RIP	Average temperature in ripening stage	Continuous	°C
DR_Avg_VEG	Average diurnal range in vegetative stage	Continuous	°C
DR_Avg_REP	Average diurnal range in reproductive stage	Continuous	°C
DR_Avg_RIP	Average diurnal range in ripening stage	Continuous	°C
TX_35_Freq_VEG	frequency of days with maximum temperature above 35°C in vegetative stage	Continuous	---
TX_37_Freq_REP	frequency of days with maximum temperature above 37°C in reproductive stage	Continuous	---
TX_31_Freq_RIP	frequency of days with maximum temperature above 31°C in ripening stage	Continuous	---
P_Accu_VEG	Accumulated precipitation in vegetative stage	Continuous	mm
P_Accu_REP	Accumulated precipitation in reproductive stage	Continuous	mm
P_Accu_RIP	Accumulated precipitation in ripening stage	Continuous	mm
P_10_Freq_VEG	Frequency of days with more than 10 mm precipitation in vegetative stage	Continuous	---
P_10_Freq_REP	Frequency of days with more than 10 mm precipitation in reproductive stage	Continuous	---
P_10_Freq_RIP	Frequency of days with more than 10 mm precipitation in ripening stage	Continuous	---
RH_Avg_VEG	Average relative humidity in vegetative stage	Continuous	%
RH_Avg_REP	Average relative humidity in reproductive stage	Continuous	%
RH_Avg_RIP	Average relative humidity in ripening stage	Continuous	%
SR_Accu_VEG	Accumulated solar energy in vegetative stage	Continuous	Cal·cm^-2^
SR_Accu_REP	Accumulated solar energy in reproductive stage	Continuous	Cal·cm^-2^
SR_Accu_RIP	Accumulated solar energy in ripening stage	Continuous	Cal·cm^-2^
Yield	Crop productivity	Continuous	Kg·ha^-1^

After the indicators were calculated, variables with low or zero variability and outliers were removed. The final datasets included 1,240 cropping events in Saldaña from 2007 to 2013, and 373 cropping events in Villavicencio from 2007 to 2014.

In order to minimize bias in the analyses, for each of the two regions, all cropping events were pooled together indifferently, independent of the year or semester.

#### 2.2.4. Observed variability for climatic variables and rice yields

Rice has been shown to be highly sensitive to climatic conditions. Yoshida [35] showed that in controlled conditions, a 100 cal·cm^-2^·day^-1^ difference in the ripening stage can result in approximately 1,000 kg·ha^-1^ impact on yield. Using observational data we had access to the wide range of climatic conditions that the crops experienced in the last seven years. Table 3 sums up the observed range for each climatic variable as well as for yield in both sites.

**Table 3:** Summary of the variability observed among all cropping events between 2007 and 2014 in each site.

	Saldaña			Villavicencio		
Variable	Minimum	Maximum	Coefficient of variation	Minimum	Maximum	Coefficient of variation
TX (°C)	23.4	39.6	0.07	23	36	0.07
TM (°C)	18.6	27.3	0.04	18	26	0.05
P_accu (mm)	115	1,229	0.36	987	1,934	0.16
P_10_Freq	0.03	0.23	0.37	0.22	0.43	0.15
RH (%)	42	95.6	0.10	61.9	96	0.07
SR_accu (cal·cm^-2^)	40,146	69,543	0.06	39,508	52,543	0.04
Yield (kg·ha^-1^)	2,000	10,750	0.21	1,750	8,200	0.29

See Table 2 for variables definitions.

The observed variability in climatic factors is attributable to climate variability as well as to the diversity of sowing dates used by rice growers. In several instances, climate characteristics reached the physiological limits of the plant. For example in Saldaña, the maximum temperatures crops were exposed to, ranged between 23.4 and 39.6°C. Optimum limits have been assessed for rice with the observation that temperatures above 37°C during anthesis may result in plant sterility [42]. SR also varied significantly: in the whole cycle, the minimum accumulated SR was 40,146 cal·cm^-2^ (daily average 391 cal·cm^-2^·day^-1^) and the maximum accumulated SR was 69,543 cal·cm^-2^ (daily average 465 cal·cm^-2^·day^-1^). In Villavicencio, total precipitation was abundant with respect to rainfed rice requirements as characterized by Fageria [43], but its distribution was variable: P_10_Freq ranged from 0.22 to 0.43 meaning that some cropping events received twice as much frequent rainfall as others. The summary of observed variability in climatic factors per growth stage is shown in S3 Table.

The dependent variable, yield, also varied significantly. Yields ranged between 2,000 and 10,750 kg·ha^-1^ in Saldaña, and between 1,750 and 8,200 kg·ha^-1^ in Villavicencio (S1 Fig). As a reference, national average yield is 6,100 kg·ha^-1^ for irrigated rice and 4,600 kg·ha^-1^ for rainfed rice [13].

With the exploratory analysis of the data confirming large variability in both yields and climatic conditions among cropping events, the following sections focus on exploring this variability to determine the relationships between yield and climatic factors at the system and cultivar levels.

### 2.3. Analysis methods

#### 2.3.1. Machine learning to detect climatic limiting factors affecting rice yield at regional scale

Machine learning techniques have become popular in many disciplines [44–46] to analyze non-experimental/observational data, as they often have lower requirements with respect to input data quality. Several algorithms used in earlier studies in similar contexts [27,44,45,47], as well as others with potentially desirable characteristics, were evaluated in relation to the main characteristics of our problem: (i) observational error and noisy data in both predictors and response variables, (ii) high likelihood of non-linear relationships between predictors and yield, (iii) high potential for correlated predictors, and (iv) the need to retrieve the relevant variables that actually explain the yield variability. We first compared algorithms in order to systematically identify those most suited to our problem (Table 4, adapted from Hastie *et al*. [48]). We then selected an implementation consistent with our analytical framework.

**Table 4:** Comparison of regression methods relative to their ability to handle different types of data problems.

	Linear Models	Neural Networks	Trees	Support Vector Machine
**Non-linear relationships**	[-] Non-linear relationships require transformation before training the model, which requires prior knowledge.	[+] Neural networks have universal approximation capabilities for non-linear relationships [49]	[+] Can model non-linear relationships.	[+] Can model non-linear relationships.
**Natural handling of “mixed” type data**	[-] Needs preliminary transformation of categorical variables.	[-] Needs preliminary transformation of categorical variables.	[+] Uses recursive binary partitions. Therefore handles categorical variables inherently.	[-] Needs preliminary transformation of categorical variables.
**Handling of missing values**	[-] Needs preliminary imputation of missing values	[-] Needs preliminary imputation of missing values	[+] Can use surrogate splits to overcome missing data.	[-] Needs preliminary imputation of missing values
**Robustness to outliers and noisy data**	[-] Typically influenced by outliers. Therefore needs preliminary filtering of such values.	[-] Known to suffer a lack of robustness towards outliers when using a classical error measure [50]	[+] Resilient to the effects of predictor outliers.	[-] One of the well-known risks of large margin training methods, such as SVMs is their sensitivity to outliers [51]
**Insensitive to monotone transformations of inputs**	[-] Typically influenced by any transformation of the inputs.	[-] Typically influenced by any transformation of the inputs.	[+] Invariant under (strictly monotone) transformations of the individual predictors.	[-] Typically influenced by any transformation of the inputs.
**Ability to deal with irrelevant inputs**	[+] Assigns a low coefficient to irrelevant inputs.	[-] Does not cope well with irrelevant input.	[+] Performs internal feature selection. Thereby resistant to inclusion of many irrelevant predictors.	[-] Does not cope well with irrelevant input.
**Interpretability**	[+] White-box model.	[-] Black-box model.	[+] Grey-box model. Tends to be white-box if the number of splits is small.	[-] Black-box model.
**Ability to deal with correlated predictors**	[-] Needs previous filtering of such correlated predictors.	[-] Needs previous filtering of such correlated predictors.	[-] Needs previous filtering of such correlated predictors.	[-] Needs previous filtering of such correlated predictors.

([+] = good, [-] = poor).

It is important to note that we do not compare the algorithms in terms of prediction power since the main purpose of this study is to explain yield variability rather than to predict it correctly. This crucial difference was highlighted by Shmueli *et al*. [52].

Given the characteristics of our data, tree-based methods are generally the best suited to our objectives. In order to reduce the variance typical of tree-based approaches, “bagging” was introduced by Breiman *et al*. [53], and works especially well for high-variance, low-bias procedures. The Random Forests algorithm (RF hereafter) [54], is a modification of the bagging approach that builds a large number of trees, and computes their average. In spite of the positive features of RF, its basic implementation suffers from several drawbacks that have been reported in former studies: it has a bias that tends to favor correlated predictors in calculating the variable importance measure (VI hereafter) and, among them, does not distinguish well the truly informative predictors from the spuriously correlated ones [55,56].

A new implementation of RF, Conditional Inference Forests (CIF), proposed by Strobl *et al*. [55], addresses these issues. CIF uses an alternative tree building process [57] with a conditional grid for the permutation importance measure, allowing better assessment of the individual contribution of each variable and better differentiation of real predictors from spurious correlations. The computed importance is then considered conditional in that a variable has a marginal importance if there is no other variable given and it partly explains the output, and a conditional importance if all other variables are given and it still brings additional explanatory value. The latter is particularly relevant for explanation tasks, and is why we used CIF.

Following the observations of Genuer *et al*. [58], and to maximize the discriminating power of the models, 2,000 trees were grown in each CIF model (ntree = 2,000), and a random sample of nine of the 27 input variables (see Table 2) was used at each node to choose the best split (mtry = p/3 = 9). An ensemble of 100 CIF models were trained for each dataset so as to mitigate the instability of the method. VI results of each model were normalized (i.e. divided by the sum of the VI scores) and scaled by the model’s performance (R-squared) to give more weight to well-adjusted models. Final VI scores of the input variables were computed as the average of the VI scores obtained by the variable over the 100 runs [48,55]. R packages party (function ctree) [57] and caret [59] were used to run the CIF models.

We performed a post-treatment of the predictors’ VI score distributions using a Kruskal-Wallis test. The multiple comparison procedure groups predictors according to their VI distribution’s position parameters, performing a significance test at 5% level (P-Value = 0.05). This enhanced the reading of the results and their interpretation by emphasizing the significant differences between VI scores distributions. This was done using the R package agricolae [60].

Models were first run per region to characterize the overall limiting factors at the regional scale. Models were then run separately for every cultivar with sufficient number of observations (n>100), in order to characterize the specific response of each cultivar to the regional climate variability. Three different cultivars were considered in Saldaña and one was considered in Villavicencio.

The computational load of CIF models is high and therefore limits the number of observations that can be analyzed in one model to approximately 500. To run the model including all the cultivars in Saldaña (n = 1,240), we followed the approach used by Nicodemus *et al*. [61] and suggested by Strobl *et al*. [55], and randomly sampled the dataset to generate 100 subsets of 400 observations. A CIF model was trained on each of the subsets, with the same parameters as above, to compute the conditional VI. Final VI of each input variable was then averaged across the 100 CIF models the same way as above.

Finally, to assess the relationships between the predictors and the output, response profiles were drawn using partial dependence plots [44,48,62]. They represent the partial relationships of the predictors to the output variable as expressed by the model.

#### 2.3.2. Clustering to classify cropping events according to their weather pattern

To further characterize the relationships of climate variability and crop yields over time, we assessed how different weather patterns influenced yields. To do this, we classified the cropping events according to their specific climatic conditions using hierarchical clustering.

Traditional clustering techniques use numeric and/or categorical variables as features to classify the individuals. In order to conserve the precision allowed by daily weather records and to enhance the clustering process, we used the whole weather patterns of the cropping events directly as features. We used the Dynamic Time Warping (DTW) distance [63–65] as a distance measure for the clustering analysis. DTW allows the direct use of the five original climatic data series that characterize each cropping event as a basis for differentiation in the clustering process. Each series holds approximately 126 daily data points, depending on the duration of the rice cropping events. This framework makes it possible to differentiate groups based on day-to-day variability, including drought and heat spells of short duration that can substantially affect rice [66,67]. The optimal number of clusters was determined based on the inertia gain in the hierarchical tree. As with VI results, we performed a Kruskal-Wallis test on clusters’ yield distributions in order to group clusters with no significant differences, and distinguish them from the others.

The clustering analyses were designed to produce groups of cropping events that experienced similar weather patterns. This would help differentiating between favorable and unfavorable patterns [67], and to get insights into each group to learn as to which cultivars performed the best under each condition. Cluster analyses were run using the R package dtw [68]. All analyses were run using the R statistical environment (R 3.1.2) [69].

## 3. Results and Discussion

### 3.1. Exploration of limiting climatic factors affecting rice yields at local scale

**Fig 2:**
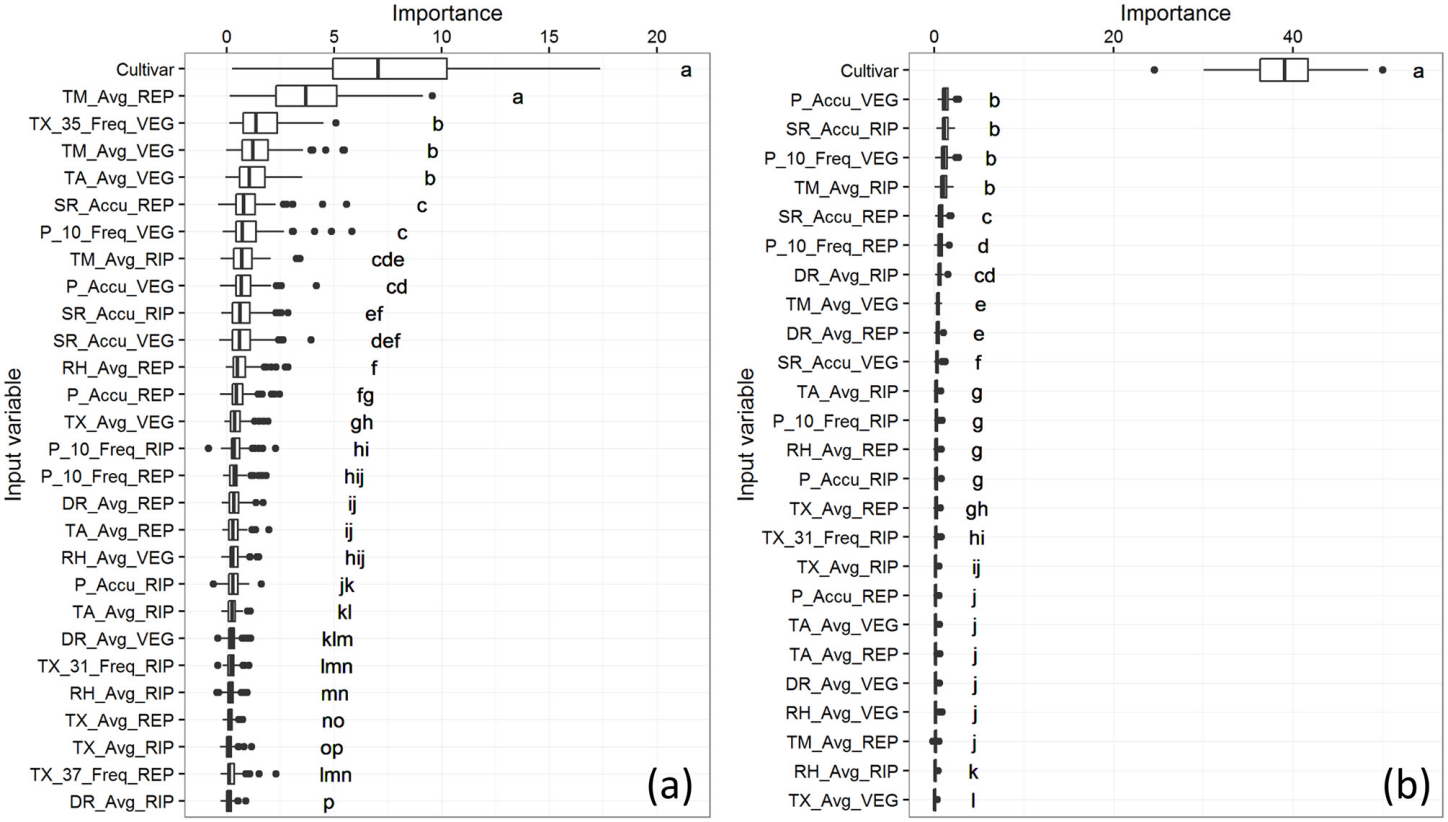
Variables importance of CIF models including all cultivars. (a) Saldaña and (b) Villavicencio. Lowercase letters to the right of the boxplots show the results of the Kruskal-Wallis test, with statistically similar variables grouped by the same letter

To gauge the general response of rice crops to climate variability in each study area we first ran models on the whole datasets with all the cultivars pooled together. The cultivar was also used as an input variable to assess its relative weight. The models yielded 26.7% and 50.2% R-squared in Saldaña and Villavicencio respectively. The R-squared obtained by each model is an indication of the fraction of the dependent variable’s (yield) variance the model could explain with the inputs [52]. Results of VI (Fig 2) illustrate the dominating role of the cultivar in explaining yield relative to the climatic factors.

The selection of the cultivar to be sown is therefore a factor of major influence on final yields in both areas.

In order to assess the importance of climatic factors in explaining yield, models were run a second time on the whole datasets but without the cultivar among the input variables. The models yielded 24.7% and 33.1% R-squared in Saldaña and Villavicencio respectively, suggesting that climatic factors alone explain approximately one quarter to one third of the yield variability. The differences in R-squared between models with and without cultivar as an explanatory variable was larger for Villavicencio than for Saldaña, suggesting that cultivar selection is of greater importance in Villavicencio than in Saldaña. This finding can be related to the difference in cropping systems, with rainfed rice (Villavicencio) being more sensitive to water availability and therefore responding more strongly the genotype by environment interactions.

In order to gain deeper insight on the responses of specific cultivars to climate variability, four region by cultivar specific models are presented in the following section: three cultivars in Saldaña (irrigated rice) and one model on the main cultivar in the rainfed system in Villavicencio. Table 5 summarizes the characteristics of the models:

**Table 5:** CIF Models inputs and results.

Model	Observations	Runs	Average R-squared	R-squared standard deviation
Saldaña—F733	267	100	29.9%	7.9
Saldaña—F60	150	100	46.6%	8.6
Saldaña—Lagunas	187	100	6.9%	5.6
Villavicencio—F174	134	100	28.1%	11.9

Models F733 and F60 in Saldaña respectively explained 29.9% and 46.6% of the yield variability which supports the hypothesis that climate variability had significant impact on these cultivars throughout the years. In turn, the Lagunas cultivar was exposed to the same climatic conditions but the model resulted in a lower R-squared. This suggests that the Lagunas cultivar was not as sensitive to climate variability as the other two. Lagunas has been reported to have good adaptability to environment (low sensitivity) [70], an observation that qualitatively coincides with the lower response of the model.

**Fig 3:**
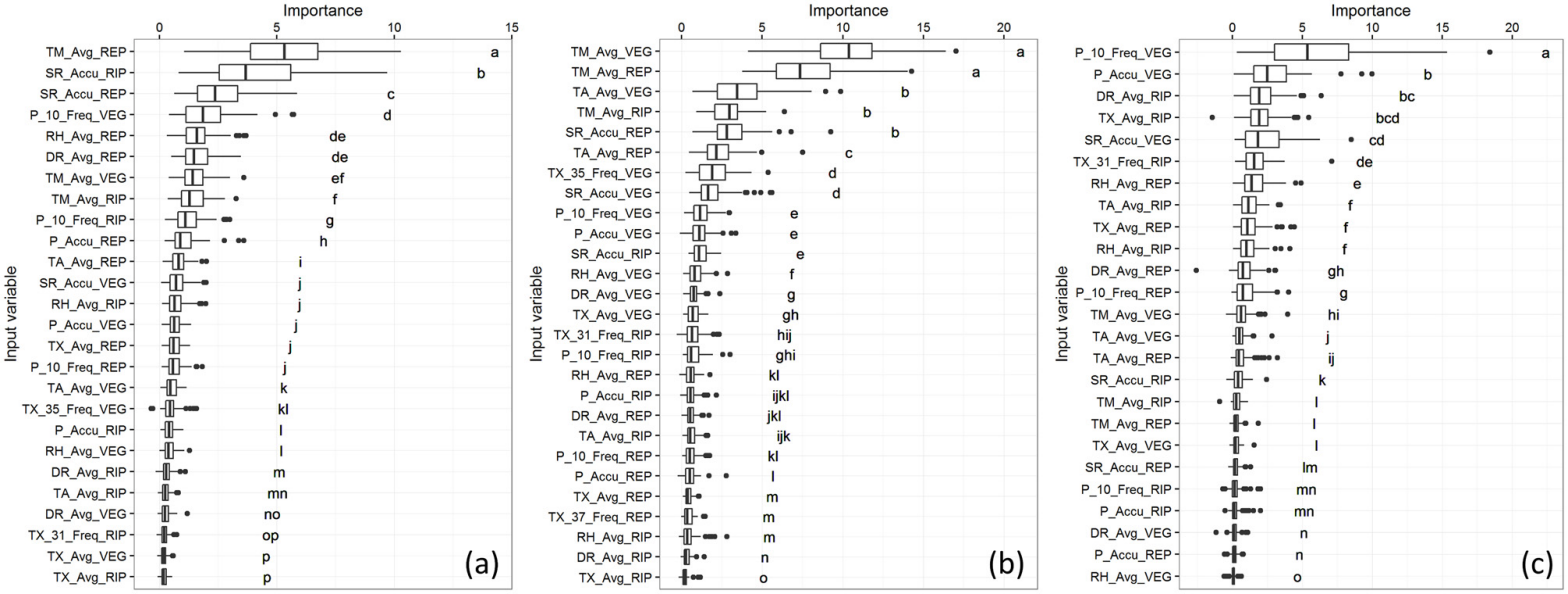
Boxplots of conditional permutation based VI scores using CIF for specific cultivars. In Saldaña for (a) cultivars F733 and (b) F60, and in Villavicencio for (c) cultivar F174. Lowercase letters to the right of the boxplots show the results of the Kruskal-Wallis test, with statistically similar variables grouped by the same letter.

**Fig 4:**
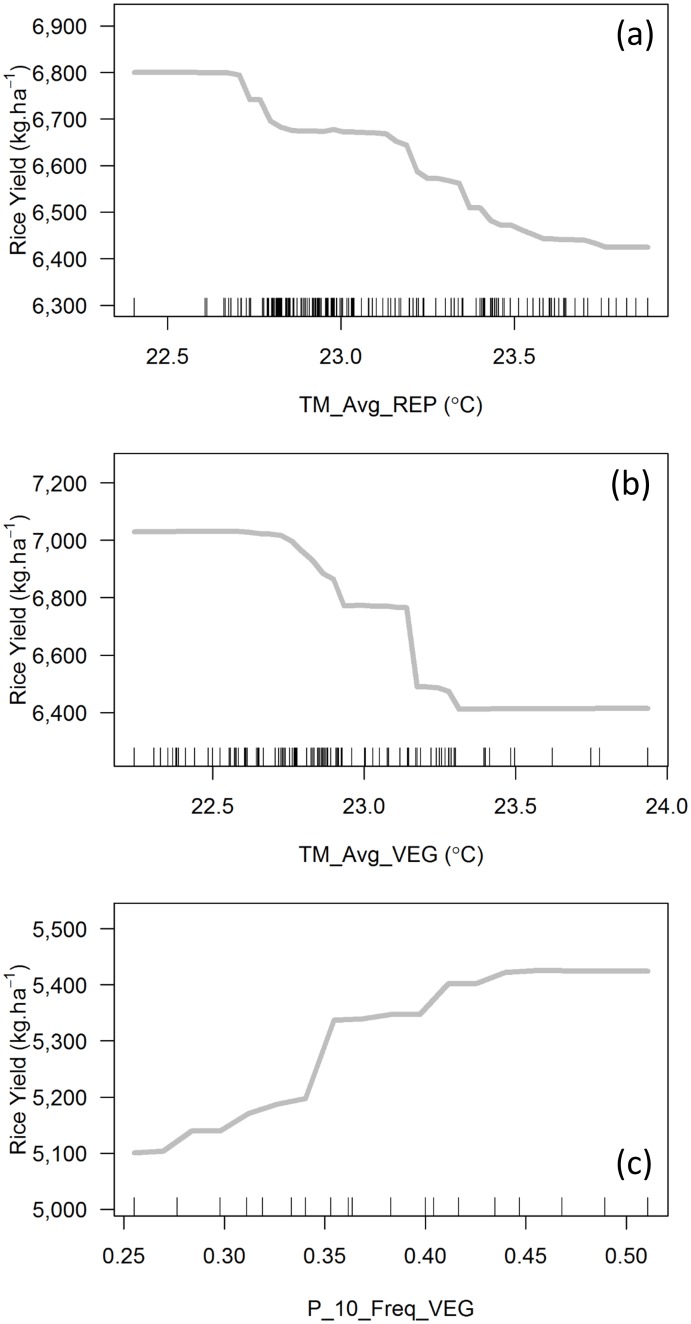
Partial dependence plots of the most relevant predictors. (a) Saldaña-F733, (b) Saldaña-F60 and (c) Villavicencio-F174. Tick marks represent individual observations.

The VI outputs for the other three models are shown in Fig 3 with the relationships between most relevant predictors and the output variable shown in Fig 4.

For Saldaña-F733 the most relevant predictor was the average minimum temperature in the reproductive stage with a gradual negative impact on yield from 22.7°C onwards (Fig 4). The negative effect of high minimum temperatures on rice yields was previously reported with similar temperature ranges [66,71]. The other two predictors with a statistically higher VI than the rest included the accumulated solar radiation in both ripening and reproductive stages, both with positive relationship to the yield (S2 Fig). This suggests that, in Saldaña, cultivar F733 tends to be limited by high night temperatures and a lack of solar radiation in the ripening stage. Sensitivity to solar radiation in the ripening stage has been reported for this cultivar [72]. Other studies conducted under controlled environments also reported this phenomenon in general for rice crops [35,73–75], as well as the combined effect with high nighttime temperatures [71].

Agricultural practices in Saldaña are not consistent with the climatology of the region. Climatological records in Saldaña emphasize two peaks of solar radiation each year. These typically occur from February to April, and from July to October. But farmers grow rice all along the year, mostly because of irrigation district constraints with rotating access to water. As a result, many rice crops are not able to exploit the optimal window of solar radiation in their last growth stage. This situation is consistent with solar radiation emerging as one of the major limiting factors. For farmers, these findings could help to open the debate on the reorganization of the irrigation district in Saldaña to allow more flexibility on sowing dates.

In the case of Saldaña-F60, the average minimum temperatures in both the vegetative and reproductive stages were the most relevant predictors (Fig 3) with a negative relationship to the crop yield (Figs 4 and S2). These results are in line with the Saldaña-F733 model outputs, with high nighttime temperatures being characteristic of Saldaña. The third most relevant predictor was the average temperature in vegetative stage with a negative effect and a breaking point at 28°C (S2 Fig), consistently pointing to high temperatures in early stages as a limitation for cultivar F60.

Finally for Villavicencio-F174, the frequency of significant rainfall (>10mm) in the vegetative stage came out as the most relevant variable (Fig 3). The relationship with yield was positive (Fig 4). This confirms the importance of water availability in rainfed rice crops, but also emphasizes that the frequency of rainfall matters more than the total amount of precipitation in this locality, especially in the vegetative stage. These results may foster the development of water harvesting and complementary irrigation infrastructure in that area to adapt to unevenly distributed rainfall.

In line with other studies in related domains, this study demonstrates that the use of empirical modelling techniques on observational data is well suited to help solving very concrete problems, and that it supports learning from data to confirm or update the knowledge of decision makers [31,76,77]. Among the existing techniques, CIF models were reported as a flexible and powerful alternative for these types of analyses [78,79].

It is noteworthy that models outputs were highly specific to each region-cultivar pair. These results confirm that cultivars respond differently to climate variability [80], and suggest that such analysis should be replicated for each local context. As in Muller *et al*. [79], we advocate for extensive reuse of the approach at local scale for agronomists and researchers to analyze their own data.

### 3.2. Understanding the relationship between weather patterns and rice yield

The dataset can be further explored to characterize the relationships between yield and weather patterns. As in section 3.1., we first explored the whole dataset in each study area to classify all the cropping events based on weather conditions, without any other criteria of differentiation. We then focused on how specific patterns affected the relative performances of individual cultivars.

**Fig 5:**
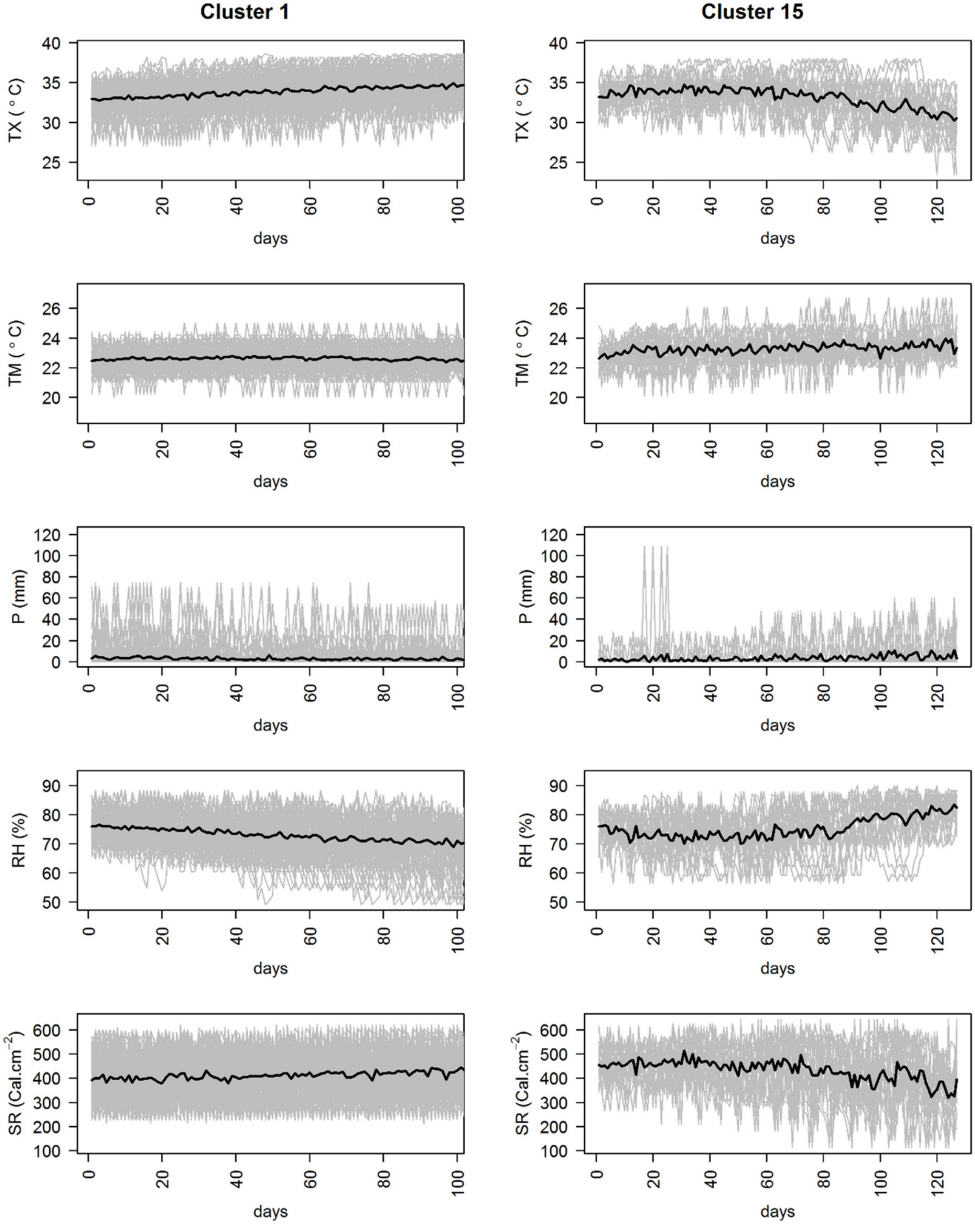
Patterns of the daily series of the 5 climatic variables characterizing the clusters. (left) Cluster 1 and (right) cluster 15. Individual patterns of each cropping event appear in grey, the black line represents the median of the cluster.

In Saldaña, we computed 15 clusters corresponding to 15 different crop-cycle long weather patterns that have occurred over the last seven years in that region. The role of the different raw series of climatic variables as the basis for the clusters is illustrated in Fig 5 where two contrasting clusters are given as examples to show the differences between them. In the highlighted example, cluster 15 shows that crops experienced low light, high nighttime temperatures, and high humidity in the late stages of the crop cycle, while the contrary was true for cluster 1.

**Fig 6:**
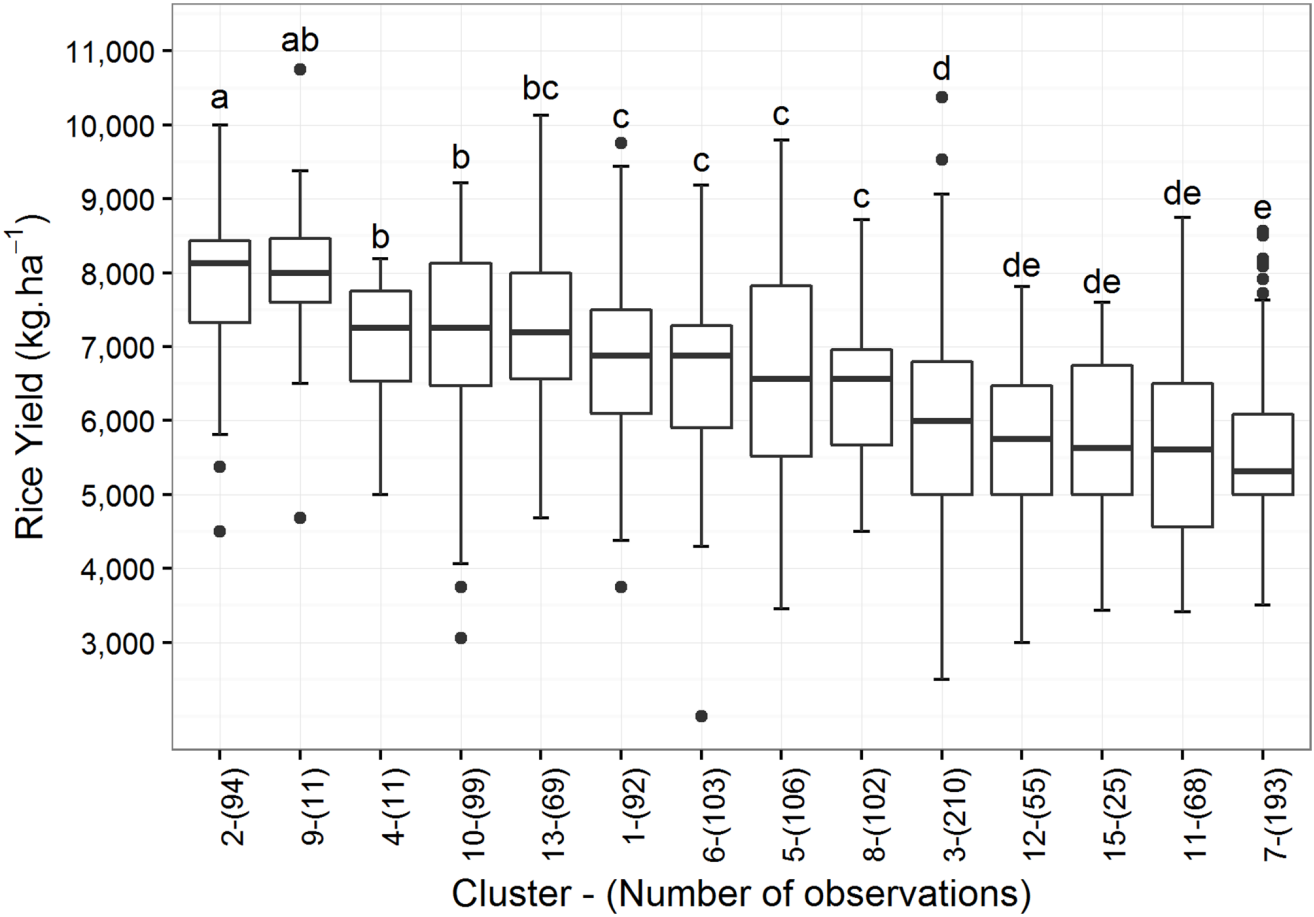
Boxplots of the observed yield distributions in each cluster in Saldaña. Clusters are sorted from left to right in decreasing order of median yield value. Lowercase letters above the boxplots show the results of the Kruskal-Wallis test, with statistically similar clusters grouped by the same letter.

The number of observations belonging to each cluster as well as the distribution of the yields achieved in each group are shown in Fig 6.

As the yield distributions observed in Fig 6 correspond to the actual yields achieved in the past for each weather pattern (cluster), they show how well rice crops (across all cultivars) responded to each weather pattern in terms of yield average and variability. Some clusters do not significantly differ in yield (1, 5 and 6 for instance) while some others differ substantially (as do cluster 2 and cluster 7).

Cluster 7 generally corresponds to events in the second semester of 2011, while cluster 2 is marked by events of the first semester of 2008, both with similar proportions of the different cultivars as compared to the whole dataset. Average yield for cluster 2 was 7,885 kg·ha^-1^, whereas for cluster 7 it was 5,659 kg·ha^-1^, and the Kruskal-Wallis test confirmed that both yield distributions differed significantly. This suggests that the weather pattern 7 was less favorable for rice cropping in Saldaña than pattern 2, which is associated with higher yields. On the other hand, it is interesting to note the differences between the patterns in term of yield variability. Under pattern 7, for example, yields were low but also showed low variability (StDev of 879 kg·ha^-1^) compared to pattern 3 where yields were highly variable (StDev of 1,327 kg·ha^-1^). For farmers, this variability in expected yields under each pattern equates to uncertainty with respect to the amount of harvested rice. Farmers have different profiles in terms of risk management, with some being able to take risks to bet on good weather and outstanding harvest while others need to guarantee a minimum level of productivity to ensure adequate income. The information generated by this cluster analysis can guide farmers in those choices. In our example, if pattern 3 were observed, low risk farmers might prefer to grow another crop in lieu rice to avoid potential crop failure.

**Fig 7:**
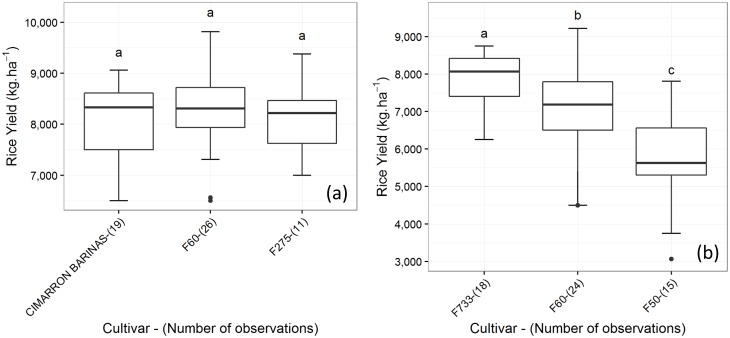
Boxplots of the yield distributions by cultivar in Saldaña. (a) Cluster 2 and (b) cluster 10. Cultivars are sorted from left to right in decreasing order of yield median value. Lowercase letters above the boxplots show the results of the Kruskal-Wallis test, with statistically similar cultivars grouped by the same letter.

Following the analysis of the complete dataset, each cluster was then analyzed separately to see if the specific cultivars had an effect on yield distributions under each pattern. Here again, in some clusters we noted a strong differentiation of the individual performance of the cultivars, while in some others, no significant differences were found. An example for each situation is shown in Fig 7; in cluster 2, no significant differences in yields, confirmed by the Kruskal-Wallis test, can be identified between the three cultivars. If climatic conditions of cluster 2 were to occur, no specific recommendations could be made regarding cultivar choice given the cultivars studied.

In contrast, cluster 10 showed a much clearer pattern. The Kruskal-Wallis test showed that significant differences exist between the distributions of observed yields of the three main cultivars: F60, F733 and F50. For this cluster, cultivar F50 resulted in low yields, whereas the yield distributions of F60 and F733 suggested that they performed much better, with F773 showing less variability than F60 and likely being the better option. When data exhibit such a clear pattern, information can be released to farmers and rural advisors: based on records associated with past experiences, some cultivars seem to be better suited than others if the specific conditions associated with cluster 10 are anticipated.

Comparable results were obtained in Villavicencio, where 10 different patterns were identified. Significant differences were detected in the yields achieved under the conditions of the different patterns (S3 Fig). Highest yields were achieved under weather patterns 2 and 7 that correspond, respectively, to sowings of April 2010 and May 2009. Yields were lower under weather patterns 3 and 4 (clusters with less than ten data points were not considered), which corresponded to sowings of 2011 and 2013. Both of these years had evenly distributed rainfall centered in June, when the crops were in the vegetative stage. Like in Saldaña, significant differences between cultivars in terms of yield performance could be detected (see cluster 4, S4 Fig). In other clusters, cultivars performed similarly.

The clustering analysis highlighted the diversity of weather patterns that rice crops can experience in both regions and demonstrated how these determine achievable yields. Studies on climate-yield relationships that use observational data often aggregate the data using either geographical or administrative boundaries [2,81]. This aggregation results in a composition effect with loss in the specificity of yield response at the farm level due to variations in sowing and harvesting dates, as well as other variables [74]. The framework illustrated here enables site-specific information to be extracted from disaggregated data, allowing climate variability to more readily be considered by farmers and agronomists who wish to make weather and site-specific recommendations on the choice of cultivar. As computational power and analysis techniques allow for handling with more and more data, future studies should avoid the aggregation of large datasets in order to exploit the full detail and information present in the disaggregated data.

### 3.3. Limitations of the study

Though the present research results in clear opportunities to improve rice agriculture in the study regions, there are aspects that can be improved. In many cases, the datasets we used did not include the original sowing dates, which we had to estimate by subtracting an averaged number of days from the harvest date. The same approach was used to estimate growth stages, with specific durations according to cultivars. This is not ideal, as the crop cycle and growth stages durations can vary due to biotic or abiotic stresses, many of them caused by climatic factors. Even if models showed little sensitivity to small changes in the dates, in future studies, datasets will need to provide measured sowing, panicle initiation and flowering dates to get a better idea of the fluctuations in crop cycle length and be able to relate them to the climate variability.

Furthermore, in this study we only considered climatic variables and this may have limited the performance of the models. In future studies, datasets that include variables for soil characteristics, climate and management should allow the models to explain greater amounts of yield variability. Finally, to improve the spatial match between crop data and weather data, geo-referenced crop data and denser weather station networks are needed.

## 4. Conclusions

The analysis of large amounts of observational crop data combined with weather records allowed us to quantify the effect of climate variability on rice. We identified the main limiting climatic factors for rice production in two localities of Colombia for a set of specific cultivars. The relatively high spatiotemporal resolution of the observational data and the use of data mining techniques allowed the assessment of weather-yield relationships with high level of detail, resulting in the ability to identify key predictor variables by growth stage. The clustering of cropping events clearly highlighted links to climate variability, with past experiences serving as collective knowledgebase for how well rice crops can perform under different weather patterns. These results give an indication of the achievable yield that can be expected under each weather pattern and, in some instances, what cultivars are best suited for each condition. Information of this type is useful for farmers to improve tactical decision making such as choosing a suitable cultivar or postponing a crop establishment to wait for a more favorable weather pattern if the current conditions are seen as less favorable.

Though we present interesting findings with regard to specific rice cultivars, the purpose of the research presented here is not to contribute new knowledge on rice. The added value of this effort is that it demonstrates how observational data can be used to efficiently generate actionable and contextualized information for on-farm decision making.

As mentioned throughout the earlier sections, our results are consistent with previous studies. Overall, the explanatory power of the models coincides with a recent worldwide study where year-to-year climate variability accounted for 32–39% of the yield variability, varying significantly depending on the region-crop combinations, and reaching up to 60% for rice in specific regions [17]. The similarity in findings further supports that the analysis of observational data using data mining techniques results in sensible information on crop response to climate variability and, likewise, supports the reliability of the approach.

The time needed for this bottom-up [82] style of data mining to understanding agricultural systems is much less than that required by more conventional experimental designs. The proposed approach can be quickly updated and the outputs can serve as part of the toolbox of agronomists and advanced farmers. It would allow them to pinpoint the specific factors that are actually limiting the productivity in their particular conditions and provide a basis for adjusting their practices accordingly.

Finally, the specificity of the response of each cultivar to the observed climate variability in the model outputs is also promising. It points towards Genotype by Environment (G*E) interaction, and suggests that the approach used in this study is useful for characterizing part of this interaction in commercial field data. Combined with the partial dependence plots of the relevant factors, this information can serve as feedback for plant breeders as it depicts actual on-farm behavior of commercial cultivars. Such feedback is a complement to mechanistic modelling for multi-site characterization of tolerances to climatic factors [83], and could guide breeding strategies towards region-specific or weather-patterns-specific cultivars [30,67,84,85].

The findings associated with the weather pattern clusters are also highly complementary to recent advances in seasonal climate forecasts, as highlighted by Meinke *et al*. [11] and Dilley *et al*. [86]. The approach presented here would be especially value added when combined with four-month seasonal forecasts, as models are now generating reliable seasonal forecasts for up to five months [7]. In comparing the forecasted four months of weather to clusters of historical records such as those derived in this study, it would be feasible to identify similarities between expected and past conditions. This would enable the use of the historical data to support improved recommendations on management practices according to successful past experiences.

The techniques implemented in the previous sections push forward the idea of data-driven agronomy as a means to positively impact agriculture by reducing the uncertainty regarding what crop to grow, where, and when to grow it. Indeed, all the information generated out of the observational data results in the expression of the collective knowledge gained from cumulative farmer experiences, and can help farmers and technical assistants to better understand the behavior of specific cultivars as well as how to better focus efforts to adapt to climate variability.

Though climate variability and climate change will continue to affect agricultural productivity, significant advances in data collection procedures, computational capacity, and improved analytics are occurring simultaneously. The use of data mining approaches on observational datasets thus represents a real opportunity to address climate variability and to bolster agricultural productivity. In order for this to be as effective as possible, however, data capture in commercial farms needs to be systematized through the use of data capture tools such as web platforms and mobile phone applications as well as remote sensing techniques.

## Supporting Information

S1 Fig Histograms of the observed rice yield distributions in Saldaña (up) and Villavicencio (down). (TIF)

S2 Fig Partial dependence plots of the relevant predictors in Saldaña. (a)(b) Saldaña-F733 and (c)(d) Saldaña-F60
(TIF)

S3 Fig Boxplots of the observed yields distributions in each cluster in Villavicencio. Clusters are sorted from left to right in decreasing order median yield value. Lowercase letters above the boxplots show the results of the Kruskal-Wallis test, with statistically similar clusters grouped by the same letter.
(TIF)

S4 Fig Boxplots of the yield distributions by cultivar under cluster 4 for Villavicencio. Cultivars are sorted from left to right in decreasing order of number of observations. Lowercase letters above the boxplots show the results of the Kruskal-Wallis test, with statistically similar cultivars grouped by the same letter.
(TIF)

S1 Table Weather stations used in Saldaña. NA stands for not available.
(DOCX)

S2 Table Weather stations used in Villavicencio. (DOCX)

S3 Table Summary of the variability observed in each growth stage in each site. See Table 2 in the manuscript for variables definitions
(DOCX)
