# Supplementary figures and images for: Assessing Weather-Yield Relationships in Rice at Local Scale Using Data Mining Approaches
Source: PLoS One. 2016 Aug 25;11(8):e0161620. doi: 10.1371/journal.pone.0161620 (PMC4999131; doi:10.1371/journal.pone.0161620)

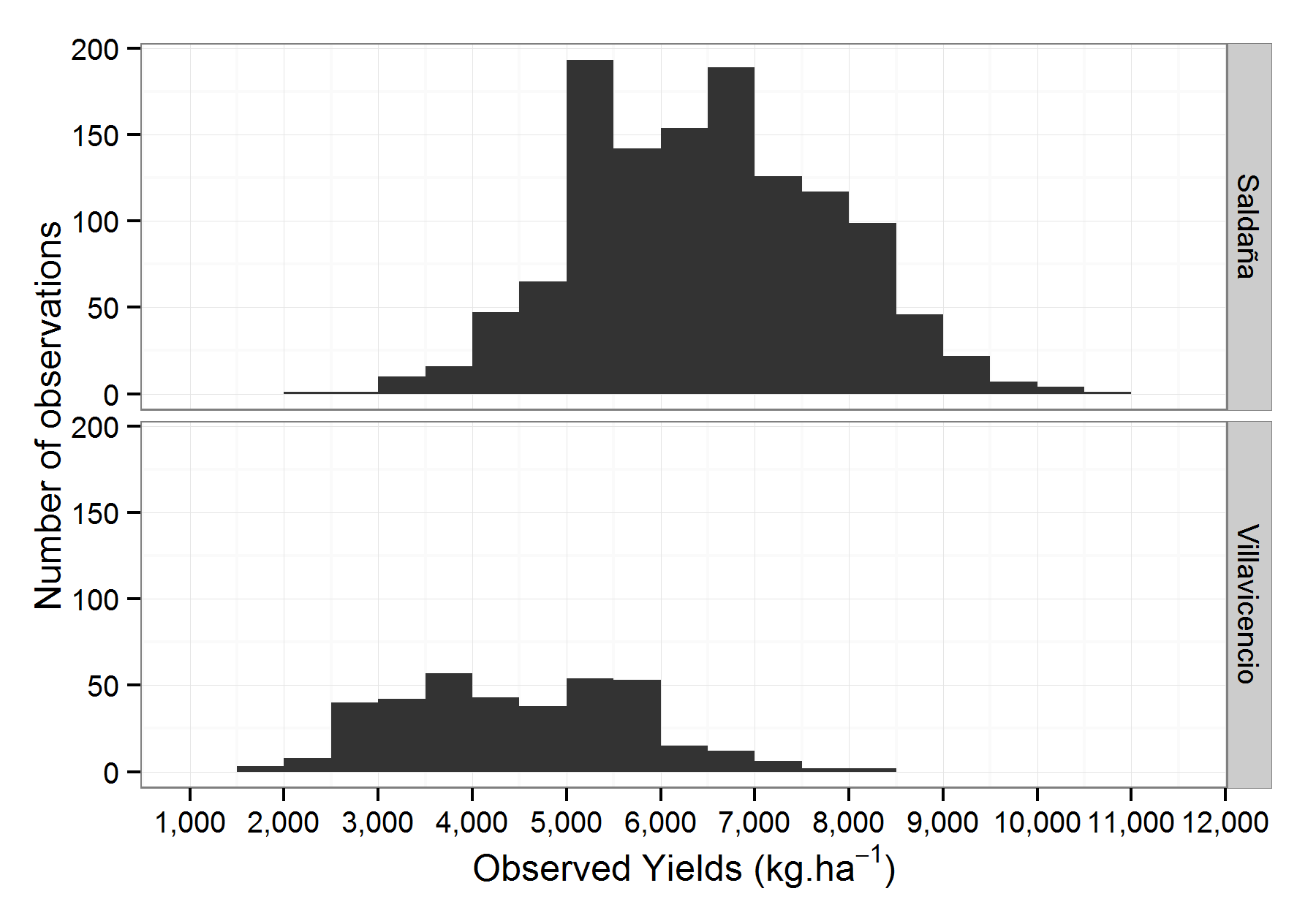

Supplement: S1 Fig — (TIF) [file pone.0161620.s001.tif]

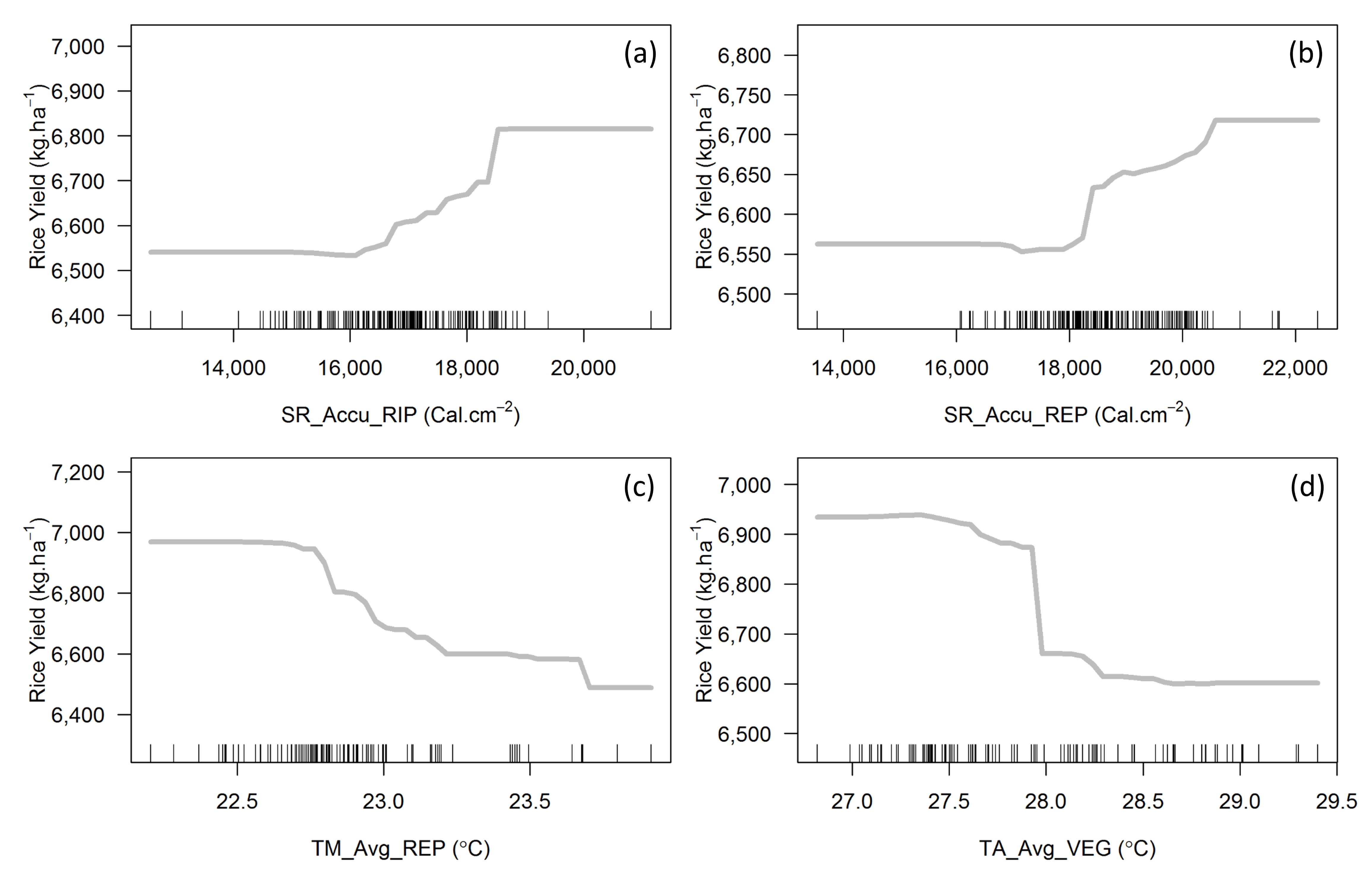

Supplement: S2 Fig — (a)(b) Saldaña-F733 and (c)(d) Saldaña-F60 (TIF) [file pone.0161620.s002.tif]

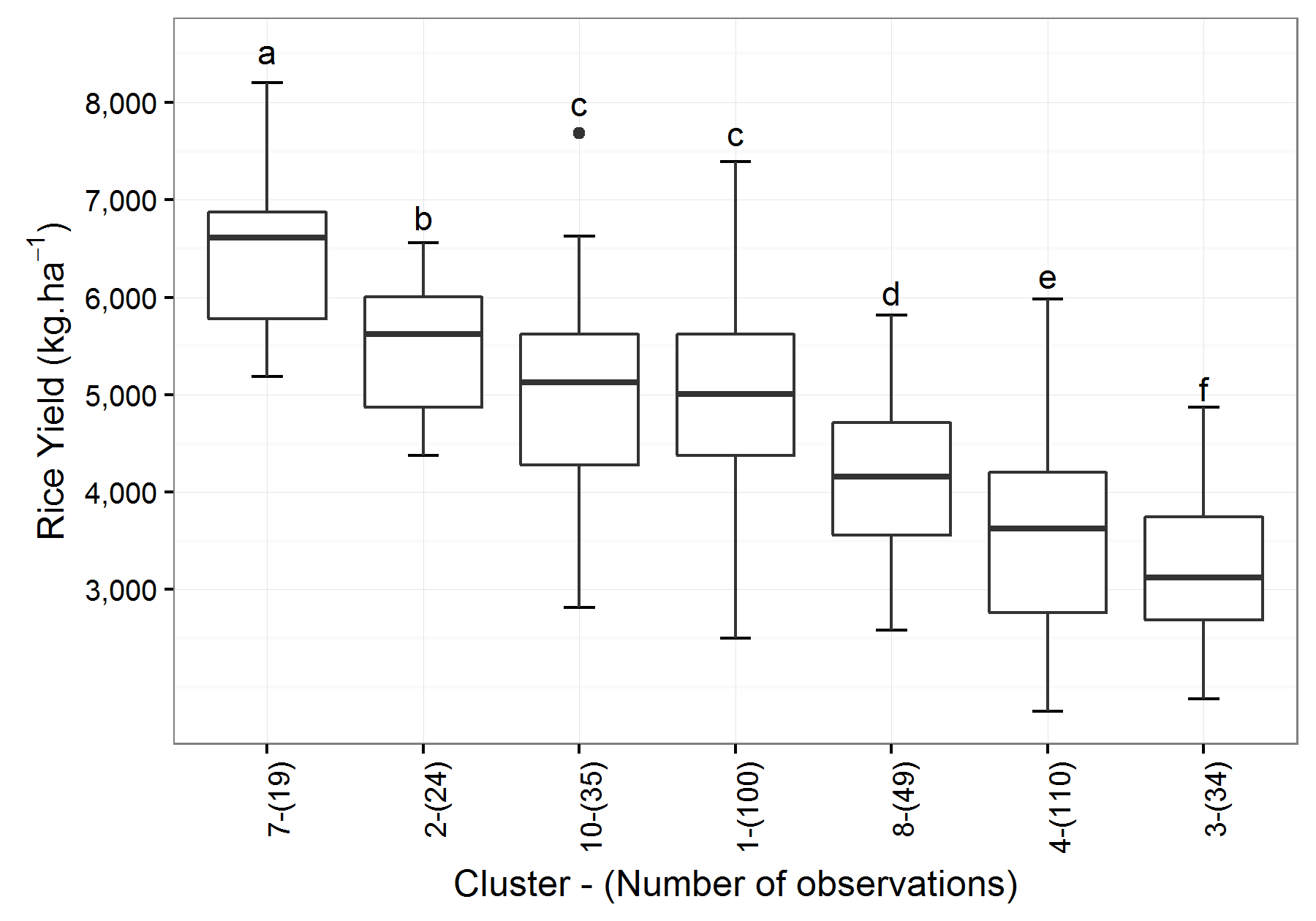

Supplement: S3 Fig — Clusters are sorted from left to right in decreasing order median yield value. Lowercase letters above the boxplots show the results of the Kruskal-Wallis test, with statistically similar clusters grouped by the same letter. (TIF) [file pone.0161620.s003.tif]

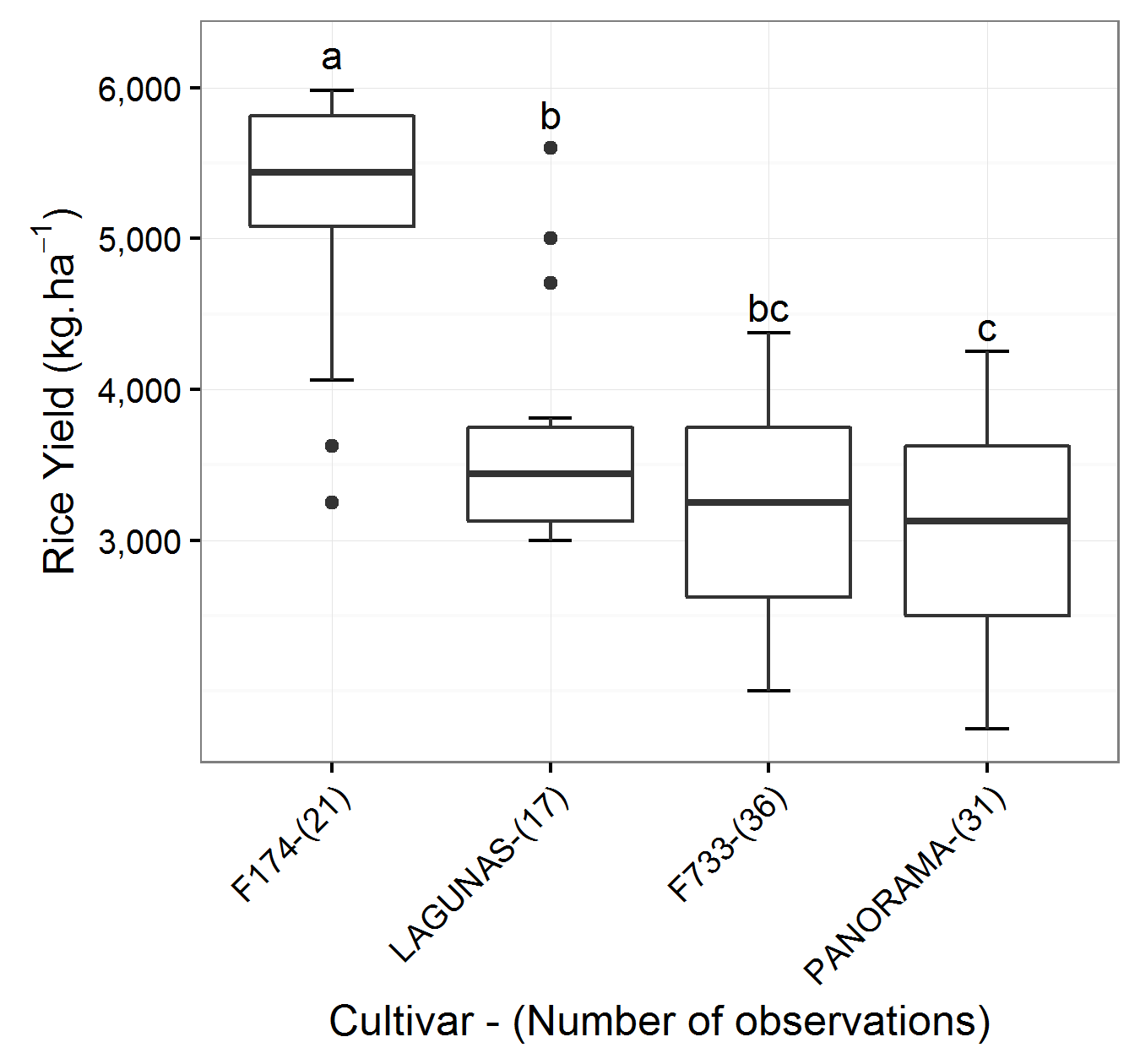

Supplement: S4 Fig — Cultivars are sorted from left to right in decreasing order of number of observations. Lowercase letters above the boxplots show the results of the Kruskal-Wallis test, with statistically similar cultivars grouped by the same letter. (TIF) [file pone.0161620.s004.tif]
