# Supplementary material for: Assessing Weather-Yield Relationships in Rice at Local Scale Using Data Mining Approaches
Source: PLoS One. 2016 Aug 25;11(8):e0161620. doi: 10.1371/journal.pone.0161620 (PMC4999131; doi:10.1371/journal.pone.0161620)
Supplement: S1 Table — NA stands for not available. (DOCX) [file pone.0161620.s005.docx]

S1 Table. Weather stations used in Saldaña.

|  | | | | | | **Proportion of missing values (%)** | | | | |
| --- | --- | --- | --- | --- | --- | --- | --- | --- | --- | --- |
| **ID** | **Owner** | **Latitude** | **Longitude** | **First record (M/D/Y)** | **Last available record (M/D/Y)** | **TX** | **TM** | **P** | **RH** | **SR** |
| 21135020 | IDEAM | 3.855777 | -75.015722 | 03/01/1964 | 04/30/2013 | 9.47 | 17.82 | 3.2 | 29.28 | NA |
| 21165010 | IDEAM | 3.787638 | -74.916944 | 03/10/1987 | 08/31/2013 | 4.62 | 3.57 | 15.2 | 22.23 | NA |
| 15 | Fedearroz | 3.913153 | -74.985072 | 06/08/2010 | 15/10/2013 | 6.19 | 6.67 | 6.91 | 7.15 | 8.76 |

NA stands for not available.
