# Supplementary material for: Assessing Weather-Yield Relationships in Rice at Local Scale Using Data Mining Approaches
Source: PLoS One. 2016 Aug 25;11(8):e0161620. doi: 10.1371/journal.pone.0161620 (PMC4999131; doi:10.1371/journal.pone.0161620)
Supplement: S2 Table — (DOCX) [file pone.0161620.s006.docx]

S2 Table. Weather stations used in Villavicencio.

|  | | | | | | **Proportion of missing values (%)** | | | | |
| --- | --- | --- | --- | --- | --- | --- | --- | --- | --- | --- |
| **ID** | **Owner** | **Latitude** | **Longitude** | **First record (M/D/Y)** | **Last available record (M/D/Y)** | **TX** | **TM** | **P** | **RH** | **Sun Bright** |
| 35025020 | IDEAM | 4.057361 | -73.467917 | 07/01/1971 | 12/31/2014 | 5.11 | 5.8 | 1 | 12.06 | 0.03 |
| 35045020 | IDEAM | 4.300444 | -73.357500 | 06/01/1961 | 01/31/2015 | 1.85 | 2.57 | 0.15 | 6.23 | 0.02 |
| 35035020 | IDEAM | 4.300444 | -73.35750 | 10/01/1924 | 02/28/2015 | 1.57 | 1.55 | 0.05 | 2.04 | 0.01 |
| 32075080 | IDEAM | 3.264989 | -73.372010 | 10/14/1990 | 10/31/2014 | 8.59 | 4.68 | 2.7 | 6.78 | 0.16 |
