# Supplementary material for: Assessing Weather-Yield Relationships in Rice at Local Scale Using Data Mining Approaches
Source: PLoS One. 2016 Aug 25;11(8):e0161620. doi: 10.1371/journal.pone.0161620 (PMC4999131; doi:10.1371/journal.pone.0161620)
Supplement: S3 Table — See Table 2 in the manuscript for variables definitions (DOCX) [file pone.0161620.s007.docx]

S3 Table. Summary of the variability observed in each growth stage in each site.

|  |  | **Saldaña** | | | **Villavicencio** | | |
| --- | --- | --- | --- | --- | --- | --- | --- |
| **Growth stage** | **Variable** | **Minimum** | **Maximum** | **Coefficient of variation** | **Minimum** | **Maximum** | **Coefficient of variation** |
| VEG | TX (°C) | 24.7 | 39.6 | 0.07 | 23.5 | 36 | 0.07 |
|  | TM (°C) | 18.6 | 27.3 | 0.04 | 18 | 26 | 0.05 |
|  | P_accu (mm) | 12 | 592 | 0.57 | 223 | 931 | 0.28 |
|  | P_10_Freq | 0.00 | 0.41 | 0.57 | 0.14 | 0.48 | 0.21 |
|  | RH (%) | 42 | 95.6 | 0.11 | 61.9 | 96 | 0.07 |
|  | SR_accu (cal.cm-2) | 13371 | 26133 | 0.07 | 11502 | 17988 | 0.11 |
| REP | TX (°C) | 24.5 | 39.6 | 0.07 | 23.5 | 34.4 | 0.06 |
|  | TM (°C) | 18.6 | 27.3 | 0.04 | 18 | 25.1 | 0.04 |
|  | P_accu (mm) | 12 | 509 | 0.54 | 146 | 900 | 0.32 |
|  | P_10_Freq | 0.00 | 0.38 | 0.55 | 0.10 | 0.50 | 0.28 |
|  | RH (%) | 42 | 95.6 | 0.11 | 69 | 95.6 | 0.06 |
|  | SR_accu (cal.cm-2) | 13544 | 24113 | 0.07 | 11080 | 16680 | 0.11 |
| RIP | TX (°C) | 23.4 | 39.6 | 0.07 | 23 | 33.9 | 0.06 |
|  | TM (°C) | 18.6 | 27.3 | 0.04 | 18 | 24.3 | 0.04 |
|  | P_accu (mm) | 2 | 529 | 0.59 | 104 | 825 | 0.37 |
|  | P_10_Freq | 0.00 | 0.42 | 0.62 | 0.09 | 0.43 | 0.35 |
|  | RH (%) | 42 | 95.6 | 0.11 | 68.1 | 95.8 | 0.06 |
|  | SR_accu (cal.cm-2) | 11739 | 21150 | 0.08 | 8502 | 21533 | 0.11 |

See Table 2 in the manuscript for variables definitions.
